# A One Health Perspective on *Aspergillus fumigatus* in Brazilian Dry Foods: High Genetic Diversity and Azole Susceptibility

**DOI:** 10.3390/jof12010072

**Published:** 2026-01-16

**Authors:** Maria Clara Shiroma Buri, Katherin Castro-Ríos, Arla Daniela Ramalho da Cruz, Thais Moreira Claudio, Paulo Cezar Ceresini

**Affiliations:** 1 Department of Crop Protection, Agricultural Engineering and Soil, São Paulo State University—UNESP, Ilha Solteira 15385-000, SP, Brazil; clara.shiroma@unesp.br (M.C.S.B.); kathecas@uis.edu.co (K.C.-R.); arla.daniela@unesp.br (A.D.R.d.C.); thais.m.claudio@unesp.br (T.M.C.); 2 Escuela de Nutrición y Dietética, Facultad de Salud, Universidad Industrial de Santander, Bucaramanga 680001, Santander, Colombia

**Keywords:** *Aspergillus*, agricultural production chain, azole resistance, microbiological surveillance, food safety

## Abstract

*Aspergillus fumigatus*, a saprophytic fungus, causes aspergillosis, primarily affecting the immunocompromised. The efficacy of triazole antifungals is compromised by resistance that has developed both clinically and environmentally. Widespread agricultural use of similar triazole fungicides selects for resistant genotypes, leading to potential food contamination and compromising treatment. This study assessed the presence of azole-resistant *A. fumigatus* in minimally processed food items commonly consumed in Brazil. A total of 25 commercial samples, including black pepper, yerba mate, and green coffee beans, were collected from different regions. Forty-two *A. fumigatus* isolates were recovered and screened for susceptibility to agricultural and clinical triazoles by determining EC_50_ values for tebuconazole (0.04–0.7 µg/mL), itraconazole (0.06–0.5 µg/mL), and voriconazole (0.07–0.15 µg/mL). Sequence analysis of the *CYP51A* gene revealed the presence of M172V mutation, none of which are associated with resistance. Microsatellite genotyping indicated high genotypic diversity and genetic relatedness among isolates from different food sources. Although no azole-resistant phenotypes were identified, the consistent recovery of *A. fumigatus* from products not directly exposed to azole fungicides highlights the need for continued surveillance. Agricultural environments remain critical hotspots for the emergence and dissemination of resistance, reinforcing the importance of integrated One Health strategies in antifungal resistance monitoring.

## 1. Introduction

Deeply rooted in history, culture, and culinary traditions, spices, teas, and coffee hold enduring significance [1]. Among them, green coffee (*Coffea*), black pepper (*Piper nigrum* L.), and yerba mate (*Ilex paraguariensis*) have become increasingly prominent in consumers’ daily lives [2,3]. These products are valued not only for their distinctive flavors and sensory properties but also for their health benefits, including antioxidant activity, anti-inflammatory effects, and metabolic support [4,5]. Amid growing interest in healthier lifestyles, demand for natural and functional foods has driven their consumption. Furthermore, green coffee, black pepper, and yerba mate hold significant socio-economic relevance in Brazil, serving as important sources of income for thousands of families and playing a key role in the national agribusiness sector [6,7].

Even though these dry products are sources of many desirable substances, they may also contain toxic metals [8,9], pesticide residues above regulatory limits [10,11,12], mycotoxins such as aflatoxin B1 and ochratoxin A [13,14,15,16], and microorganisms harmful to health [17,18]. Dry food products, such as spices, teas, and coffee beans, are often contaminated with high levels of microorganisms [19]. This contamination can occur at various stages of the production chain and may involve bacteria, viruses, and fungi, posing significant health risks, particularly when these products are used in the preparation of ready-to-eat foods [20].

Among pathogenic fungi, the genus *Aspergillus* stands out for its xerophilic and thermotolerant species, which thrive in low-moisture and high-temperature environments. Some of these species not only produce mycotoxins but can also act as opportunistic pathogens in humans, as is the case with *Aspergillus fumigatus* [21,22]. The clinical importance of *A. fumigatus* lies in its ability to cause a wide range of respiratory conditions, from localized airway inflammation to severe infections, notably invasive aspergillosis (IA). Although this cosmopolitan fungus is not typically a primary human pathogen, it has emerged as a significant opportunistic agent, particularly due to the increasing prevalence of conditions that require immunosuppressive therapies, including but not limited to cancer and organ transplantation [23]. Aspergillosis is an environmentally acquired respiratory disease that encompasses a spectrum of clinical and radiological manifestations, determined mainly by the host’s immune status [24]. It is estimated that approximately 4.8 million people worldwide are affected by some form of aspergillosis, with an estimated 300,000 cases of IA occurring annually, and associated mortality rates ranging between 30% and 95% [25,26,27].

The first-line treatment for aspergillosis relies primarily on triazole-based antifungal agents, with itraconazole, voriconazole, and posaconazole being the most commonly used [28]. However, increasing reports of reduced efficacy have been linked to the emergence of triazole-resistant *A. fumigatus* strains. These resistant isolates often harbor mutations in the *CYP51* gene, which encodes lanosterol 14α-demethylase, an essential enzyme in the ergosterol biosynthesis pathway that produces the primary sterol component of the fungal cell membrane [29,30]. Furthermore, resistance mechanisms to triazoles in *Aspergillus* spp. can be broadly categorized into two types: those associated with *CYP51A* alterations and those that are independent of this gene [31].

*CYP51A*-related resistance mechanisms are the most widespread globally [32]. Since their first identification in 1998 at a hospital in the Netherlands, an increasing number of mutations in the *CYP51A* gene have been reported. These alterations have been found in *A. fumigatus* isolates from patients undergoing long-term triazole therapy, as well as from triazole-naïve patients and environmental samples [33,34]. Mutations within the coding sequence of the *CYP51A* gene, such as TR34/L98H, TR34/L98H/S297T/F495I, TR46/Y121F/T289A, and TR53, are commonly associated with pan-azole resistance, conferring reduced susceptibility to all triazole antifungals [35]. Additionally, certain amino acid substitutions, including M220, G54, and G138, have been shown to impair drug–target interaction by diminishing the binding affinity between the antifungal agent and the fungal enzyme [36,37]. In contrast, other substitutions, such as F46Y, M172V, and E427K, are typically associated with lower levels of azole resistance [38]. The detection of TR34/L98H and TR46/Y121F alleles in both environmental and clinical isolates of *A. fumigatus* suggests that *CYP51A* mutations may arise not only in response to prolonged antifungal therapy, but also as a consequence of triazole fungicide application in agriculture, given the structural similarity between medical and agricultural triazoles [39,40]. Although the use of such fungicides is restricted in certain crops—such as black pepper and yerba mate, which are the focus of our investigation—adjacent areas under intensive agricultural practices may act as resistance hotspots. Strains selected under fungicide pressure in these regions can subsequently contaminate nearby crops at various points in the production chain. Moreover, the study by Cui et al. [41] demonstrated that pre-exposure to the agricultural fungicide tebuconazole in *A. fumigatus* isolates can induce cross-resistance to itraconazole and voriconazole. Therefore, the emergence of azole-resistant *A. fumigatus* strains in the environment should be regarded as an unintended consequence of triazole use in agriculture, especially considering that this fungal species is not a plant pathogen. Although *A. fumigatus* strains resistant to triazoles have been isolated from soil, plant debris, air, and compost samples [40,42], only the study by Viegas et al. [43] reported isolates from the Fumigati section resistant to posaconazole in tea samples. Nonetheless, this study did not investigate potential mutations associated with the observed resistance profile. Yet, *A. fumigatus* is frequently found in food products, posing a potential risk [44].

This scenario underscores the critical need for a “One Health” perspective, defined by the One Health High-Level Expert Panel (OHHLEP) as a collaborative, multisectoral, and transdisciplinary approach that recognizes the inseparable interconnection among human, animal, and environmental health [45]. This framework operates on the premise that the well-being of people is dependent on functioning ecosystems and a healthy environment [46]. Regarding antifungal resistance, this approach reveals that clinical treatment failure is not isolated from agricultural practices, as environmental resistance directly influences clinical outcomes [38,47]. The widespread use of agricultural fungicides structurally similar to medical azoles creates environmental selective pressure [39,40]. Consequently, agricultural fields can act as a potential hotspots for the emergence of resistant genotypes, which may subsequently contaminate food products and disperse to susceptible human hosts [41,48]. Understanding this environmental route of transmission is essential for mitigating the global threat of antifungal resistance.

Accordingly, this study aimed to investigate the resistance status of *A. fumigatus* isolates sampled from commercial yerba mate, black pepper, and green coffee products to the medical azoles itraconazole and voriconazole, and to the agricultural azole tebuconazole. We compared the phenotypic and genotypic profiles of fungicide susceptibility. Additionally, we performed sequencing of genes encoding fungicide target proteins, and microsatellite typing based on short tandem repeats (STRAf) was conducted for all strains to assess genetic relatedness and identify potential clonal expansion, thereby establishing a critical baseline for these specific commodities.

## 2. Materials and Methods

### 2.1. Sampling and Strain Isolation of A. fumigatus

A total of 25 dried product samples available on the Brazilian market were included in this study, as detailed in Table 1. The samples were collected between 2021 and 2022, were within their shelf life, and had been stored under proper conditions (Figure S1).

The morphological identification and isolation of fungal colonies were performed using the direct plating technique for whole-grain samples and the surface plating technique for ground and chopped samples (Figure S2), as recommended by Pitt and Hocking [50] for the analysis of food samples aimed at fungal detection. For direct plating, the whole grains were placed onto potato dextrose agar (PDA, Kasvi, Gujarat, India) supplemented with chloramphenicol. For the alternative method, ground and chopped food samples were diluted in peptone water (Kasvi, India) to 10^−3^. Then, 0.1 mL of the resulting suspension was transferred onto the surface of the PDA medium. The samples were subsequently incubated at 37 °C for 5–7 days. Once mycelial growth was observed on the Petri plates, fungal mycelium fragments were transferred to new PDA plates (subculturing) and incubated under the same conditions.

Following incubation, individual fungal colonies were transferred to malt extract agar (MEA) and incubated at 37 °C for 48 h for preliminary genus-level identification based on phenotypic characteristics, as described by Pitt and Hocking [50]. Colonies exhibiting macroscopic and microscopic features consistent with *A. fumigatus* were preserved on 0.5 cm diameter filter paper discs in cryotubes containing silica gel and stored at −20 °C [51].

### 2.2. PCR Amplification for Species Confirmation

To identify the selected isolates at the species level, a PCR assay was performed. Genomic DNA was extracted from lyophilized mycelium grown on PDA medium for 72 h at 37 °C using the Wizard^®^ DNA purification kit (Promega, Madison, WI, USA). DNA was quantified using a Nanodrop^®^ 2000c spectrophotometer (Thermo Fisher Scientific, Waltham, MA, USA). Identification of *A. fumigatus* was performed by PCR using *β-tubulin* and *rodletA* primers specific for *Aspergillus* section Fumigati and *Aspergillus fumigatus* (Table 2). Amplifications were carried out in a ProFlex PCR thermal cycler (Applied Biosystems, Waltham, MA, USA) under the following cycling conditions: After an initial denaturation at 95 °C for 5 min, amplification was performed in a total of 35 cycles of denaturation at 94 °C for 30 s, annealing at 48 °C for 30 s, extension at 72 °C for 90 s, and a final extension at 72 °C for 7 min.

### 2.3. Fungicide Sensitivity Testing

For the phenotypic assessment of susceptibility to fungicides and triazole antifungals, a total of nine *A. fumigatus* isolates were selected. These isolates were recovered from different sources: two from ground black pepper, three from whole black pepper, two from yerba mate, and two from green coffee beans.

Fungicide sensitivity tests were conducted using tebuconazole and the antifungal agents itraconazole and voriconazole (all from Sigma-Aldrich, Burlington, MA, USA), adapted from the methodology described by Brackin et al. [53]. Tebuconazole was dissolved in DMSO to prepare a stock solution at 50 µg mL^−1^, while itraconazole and voriconazole were dissolved in deionized water to obtain stock solutions at 10 µg mL^−1^. To achieve the final working concentrations, stock solutions were mixed with PDA medium supplemented with chloramphenicol at 50 µg mL^−1^, resulting in the following concentrations: tebuconazole at 0, 0.03, 1, 2, 4, and 8 µg mL^−1^; itraconazole at 0, 0.03, 1, 2, 4, and 8 µg mL^−1^; and voriconazole at 0, 0.03, 0.12, 0.25, 0.5, and 1 µg mL^−1^. Sensitivity assays were performed in 60-mm-diameter Petri dishes (see Figures S3–S5). Mycelial fragment suspensions for the sensitivity tests were prepared from fungal colonies cultured on PDA for 72 h at 37 °C. The suspensions were obtained by adding 5 mL of sterilized deionized water containing 0.05% (*v*/*v*) Tween-20 to the fungal cultures. After five minutes, 5 µL of the resulting suspension were carefully pipetted onto PDA medium containing the respective fungicide concentrations. The Petri dishes were sealed with plastic film to prevent drying and external contamination and incubated at 37 °C for 48 h, after which fungal growth was measured using a digital caliper. The experimental design followed a completely randomized block structure with four replicates per treatment, and all experiments were conducted in duplicate. Sensitivity to triazoles was evaluated by determining the effective concentration required to inhibit 50% of fungal growth (EC_50_, in µg mL^−1^), estimated using the ec50estimator package in RStudio (version 2025.09.0) in conjunction with the drc package for dose–response curve analysis. Analysis of variance (ANOVA) and mean comparisons were performed using the Scott-Knott test at a 5% significance level, implemented with the agricolae and ScottKnott packages in the R environment.

### 2.4. Genetic Diversity and Presence of Triazole-Resistant Alleles in A. fumigatus Sampled Black Pepper, Yerba Mate, and Green Coffee

#### 2.4.1. Analysis of the Allelic Variation in the CYP51A Gene from *A. fumigatus*

To identify resistance mechanisms associated with allelic variation in the *CYP51A* target gene for triazole fungicides, the methodology of Fraaije et al. [54] was applied. The specific primer sequences used for amplification and sequencing are listed in Supplementary Table S1 [54]. PCR reactions were carried out in a final volume of 25 µL, comprising ultrapure water, 25 ng of total DNA, 0.3 µM of each primer, 0.2 mM of each dNTP, 2 mM MgCl_2_, 2.5 µL of 10× buffer, and 1 unit of Taq DNA polymerase (Sigma-Aldrich). Amplifications were conducted using a ProFlex thermal cycler (Applied Biosystems) under the following cycling conditions: initial denaturation at 95 °C for 5 min, 40 cycles of denaturation at 95 °C for 30 s, annealing at 55 °C for 3 min, and extension at 72 °C for 2 min, with a final extension step at 72 °C for 8 min. PCR products from *A. fumigatus* isolates were sequenced using a PE Applied Biosystems ABI-3730 sequencer (Macrogen Inc., Seoul, Republic of Korea).

#### 2.4.2. STRAf Genotyping of *A. fumigatus*: Microsatellites Based on Short Tandem Repeats

Fungal isolates were genotyped using nine microsatellite (STR) markers [55], detailed in Supplementary Table S2. Forward primers were designed to contain a 5′-TGTAAAACGACGGCCAGT-3′ (M13F) tail at the 5′ position following the procedures described by Schuelke [56]. Four M13F primers were individually labeled with one of the fluorescent dyes 6-FAM, TET, CY3 or CY5 (Exxtend biotechnology, Paulínia, São Paulo, Brazil). The PCR reactions were performed separately for each STRAf locus in a final volume of 25 μL. Each reaction contained 5 μL of genomic DNA (final concentration 25 ng μL^−1^), 12.50 μL of GoTaq Green Master Mix (Promega, USA), 0.3 mM of each forward-M13F tailed and reverse STRAf primers, and 0.3 mM of fluorophore-labeled M13F. For all sets of primers, the PCR program included an initial denaturation at 95 °C for 5 min followed by 35 cycles of denaturation at 95 °C for 30 s, annealing at 60 °C for 35 s, and extension at 72 °C for 1 min, with a final extension at 72 °C for 8 min. Fragment analysis of the amplified PCR products was performed by Macrogen Inc., Republic of Korea, using an ABI 3700 sequencer (Applied Biosystems). The electropherogram data were analyzed in Geneious Prime version 6.7.1 (Biomatters, Auckland, New Zealand) to determine the sizes of the PCR products. Genotypic data were obtained by considering different PCR product sizes as different alleles at each locus.

Genotypic analyses of *A. fumigatus* isolates were performed using RStudio (version 2025.09.0), with emphasis on the ‘poppr’ and ‘adegenet’ (version 2.9.8) packages. Microsatellite genotypes were imported from a GenAlEx-formatted dataset and converted into genind and genclone objects for analysis. Missing data were imputed using the most common allele within each multilocus genotype.

The number of unique multilocus genotypes (MLGs) and their frequencies were calculated using mlg.table and related functions. Population subdivision was defined by the origin of the food source (green coffee, yerba mate, whole black pepper, ground black pepper).

To assess genetic relatedness among MLGs, Bruvo’s distance was calculated using repeat lengths specific to each microsatellite marker. A minimum spanning network (MSN) was then constructed with bruvo.msn(), and visualized using plot_poppr_msn(). Node sizes were scaled by MLG frequency, and populations were distinguished by color according to food source. Figures were generated with ggplot2 and patchwork, while network plots relied on igraph-based visualizations.

## 3. Results

The results obtained from the phenotypic susceptibility analysis and genotypic characterization of the *A. fumigatus* isolates recovered from the food samples are presented below.

From the 25 samples analyzed, a total of 208 fungal isolates were recovered, of which 42 were confirmed as *A. fumigatus*. All confirmed isolates were analyzed for *CYP51A* gene variation and microsatellite-based genotypic characterization. However, only nine isolates were randomly selected for antifungal susceptibility testing, as the validated phenotyping methodology is highly labor-intensive [53].

### 3.1. Susceptibility to Triazoles

**Figure 1:**
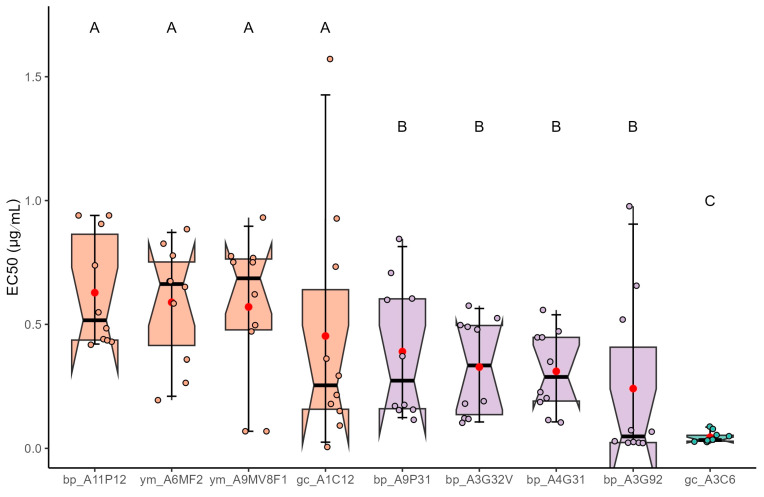
EC_50_ estimates and Scott-Knott grouping indicating reduced sensitivity of *A. fumigatus* to tebuconazole in different dried plant products (black pepper (bp), green coffee (gc), yerba mate (ym). Different letters/colors indicate significant differences (Scott-Knott, *p* < 0.05); circles show individual data points.

The median effective concentrations (EC_50_) of *A. fumigatus* isolates obtained from black pepper, green coffee, and yerba mate samples were determined in response to the three triazole fungicides tested. For tebuconazole, EC_50_ values (Figure 1) ranged from 0.0436 to 0.7098 µg/mL, reflecting variations in susceptibility among isolates from different sources. Isolates from black pepper exhibited EC_50_ values ranging from 0.0666 to 0.6039 µg/mL. The lowest values were observed for isolates bp_A3G92 and bp_A3G32V, while bp_A1IP12 presented one of the highest values within this group. Green coffee isolates ranged from 0.0436 to 0.4151 µg/mL, with gc_A3C6 exhibiting the lowest EC_50_. Yerba mate isolates showed values of 0.7098 µg/mL (ym_A6MF2) and 0.6068 µg/mL (ym_A9MV8F1), representing the upper end of the observed range.

Statistical analysis using the Scott-Knott clustering test revealed significant differences (*p* < 0.05) in tebuconazole susceptibility among the *A. fumigatus* isolates. Isolates bp_A1IP12, ym_A6MF2, ym_A9MV8F1, and gc_A1C12 were grouped in category A, indicating the highest mean EC_50_ values. In contrast, isolate gc_A3C6 was classified alone in category C, showing the lowest mean EC_50_ and, consequently, the greatest susceptibility to tebuconazole. The remaining isolates were assigned to category B, with intermediate mean EC_50_ values. The difference between the extreme groups (A and C) highlights a statistically significant variation in tebuconazole efficacy among isolates from different sources, with a mean EC_50_ difference of approximately 16-fold.

**Figure 2:**
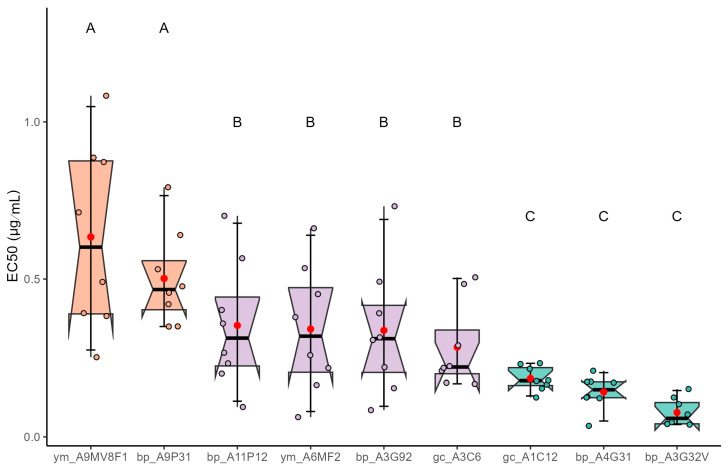
EC_50_ estimates and Scott-Knott grouping indicating reduced sensitivity of *A. fumigatus* to itraconazole in different dried plant products (black pepper (bp), green coffee (g), yerba mate (ym). Different letters/colors indicate significant differences (Scott-Knott, *p* < 0.05); circles show individual data points.

For itraconazole, the median effective concentrations (EC_50_) of *A. fumigatus* isolates exposed varied according to the source. EC_50_ values ranged from 0.0687 to 0.5872 µg/mL, indicating heterogeneity in susceptibility (Figure 2). Isolates from black pepper exhibited values ranging from 0.0687 to 0.4885 µg/mL, with bp_A3G32V being the most susceptible and bp_A9P31 the least. In green coffee, EC_50_ values ranged from 0.1799 to 0.2560 µg/mL, while isolates from yerba mate showed higher values of 0.3082 and 0.5872 µg/mL, suggesting reduced susceptibility in this group.

Statistical analysis using the Scott-Knott grouping test revealed significant differences (*p* < 0.05) in the susceptibility of *A. fumigatus* isolates to itraconazole. Isolates ym_A9MV8F1 and bp_A9P31 were grouped in category A, exhibiting the highest mean EC_50_ values, indicative of reduced susceptibility. In contrast, isolates gc_A1C12, bp_A4G31, and bp_A3G32V were assigned to category C, showing the lowest EC_50_ values and thus the greatest sensitivity to itraconazole. The remaining isolates were classified into category B, with intermediate susceptibility. The difference between the most and least susceptible groups (categories A and C) is statistically significant, with a maximum difference of approximately 8.5-fold in mean EC_50_ values (0.0687 to 0.5872 µg/mL), reinforcing the heterogeneity in response among isolates from different plant sources.

**Figure 3:**
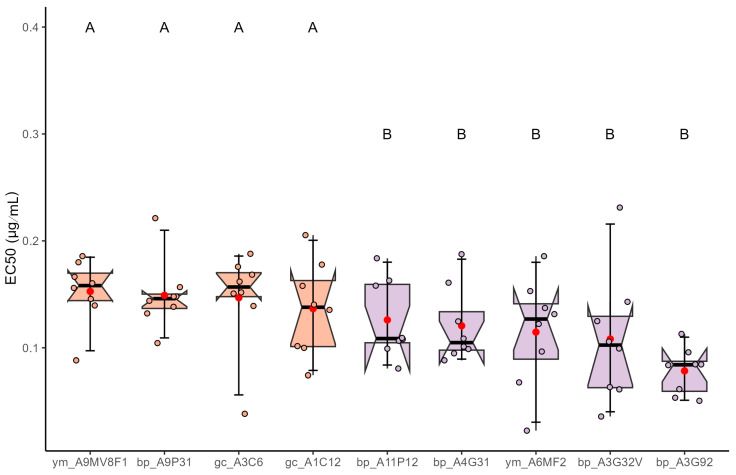
EC_50_ estimates and Scott-Knott grouping indicating reduced sensitivity of *A. fumigatus* to voriconazole in different dried plant products (black pepper (bp), green coffee (g), yerba mate (ym). Different letters/colors indicate significant differences (Scott-Knott, *p* < 0.05); circles show individual data points.

Susceptibility to voriconazole showed less variability; the median effective concentrations (EC_50_) of *A. fumigatus* isolates exposed ranged from 0.0779 to 0.1541 µg/mL (Figure 3). Isolates obtained from black pepper exhibited EC_50_ values between 0.0779 (bp_A3G92) and 0.1523 µg/mL (bp_A9P31). Green coffee isolates ranged from 0.1373 to 0.1541 µg/mL, while yerba mate isolates ranged from 0.1349 to 0.1523 µg/mL. These results indicate only slight variability in susceptibility among the isolates, with all EC_50_ values remaining well below the MIC_50_ threshold established for susceptible strains, supporting their classification as voriconazole-sensitive.

Statistical grouping using the Scott-Knott test revealed limited but significant differences (*p* < 0.05) in voriconazole susceptibility among *A. fumigatus* isolates. Isolates ym_A9MV8F1, bp_A9P31, gc_A3C6, and gc_A1C12 were assigned to group A, showing the highest mean EC_50_ values within a narrow range (approximately 0.1349–0.1541 µg/mL). In contrast, isolates such as bp_A11P12, bp_A4G31, bp_A3G92, bp_A3G32V, and ym_A6MF2 were assigned to group B, with slightly lower EC_50_ values (values ranged from 0.0779 to 0.1281 µg/mL). Although the differences between groups A and B were statistically significant, the overall variation in EC_50_ values was relatively small—approximately 2-fold—and all values remained well below the MIC_50_ threshold for resistant strains. This indicates a general susceptibility to voriconazole across all isolates tested.

### 3.2. CYP51A Gene Analysis

Sequence analysis of the *CYP51A* gene, the target for triazole-based fungicides, revealed a non-synonymous polymorphism in the *A. fumigatus* isolate bp_A3G32V, characterized by a methionine-to-valine substitution at codon 172 (M172V). No other mutations commonly associated with triazole resistance were identified in the analyzed isolates.

### 3.3. Genetic Diversity and Population Structure

**Figure 4:**
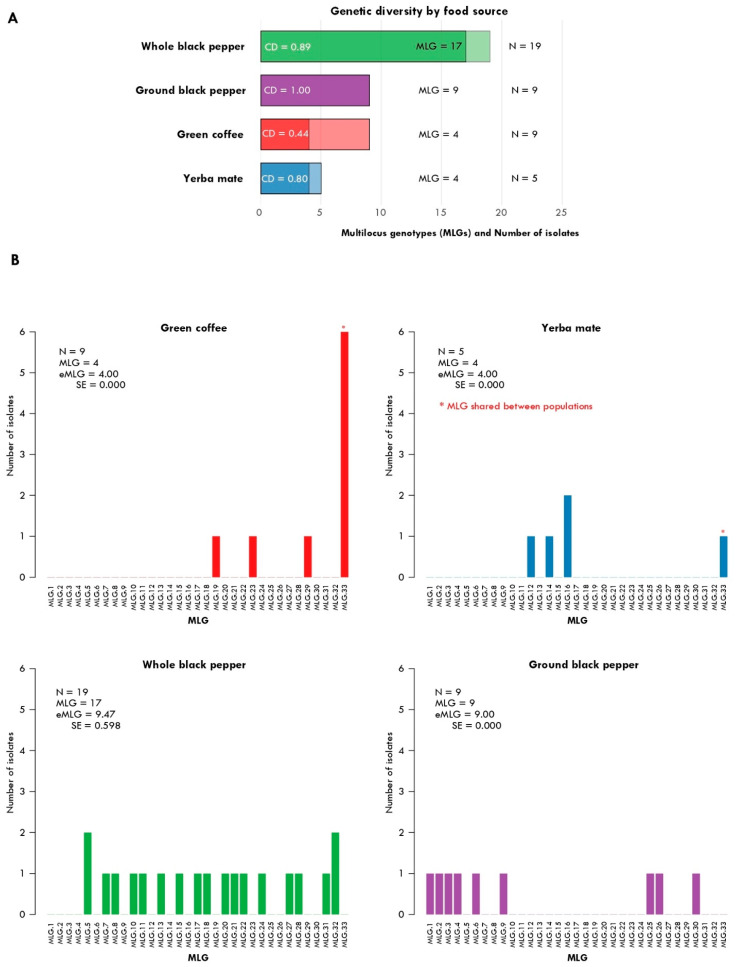
Genotypic diversity summary and MLG frequencies. (**A**) Summary of total isolates and unique MLGs per substrate, with labels indicating counts and genotypic diversity (MLG/isolates) for each population. Stacked barplots show the number of *Aspergillus fumigatus* isolates (light shade) and the corresponding number of unique MLGs (dark shade) detected in each food source. Substrates include green coffee, yerba mate, whole black pepper, and ground black pepper. (**B**) Distribution of MLGs within each substrate, displayed as barplots of MLG frequencies. Each facet represents one food source, showing the relative dominance or evenness of MLGs across populations.

A total of 42 isolates of *A. fumigatus* collected from four food sources (green coffee, yerba mate, whole black pepper, and ground black pepper) were successfully genotyped using eight microsatellite loci. Analysis revealed 33 unique multilocus genotypes (MLGs). The distribution of isolates and MLGs varied among substrates (Figure 4A). Green coffee yielded the highest number of isolates but showed moderate genotypic diversity. In contrast, ground black pepper, despite fewer isolates, exhibited a higher proportion of unique MLGs, indicating elevated genotypic diversity. Yerba mate and whole black pepper populations presented intermediate patterns, with some MLGs represented multiple times across samples.

**Figure 5:**
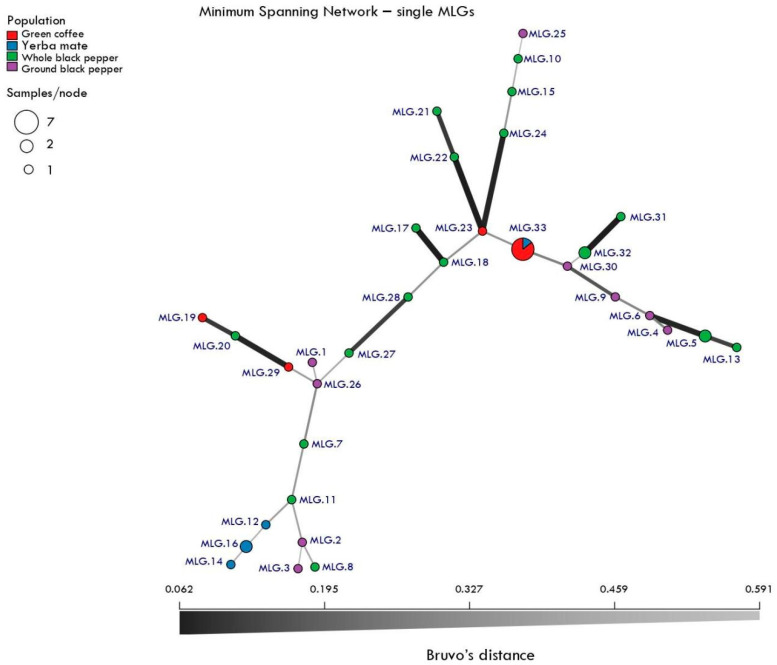
Minimum spanning network (MSN) of multilocus genotypes. Network of 33 MLGs based on Bruvo’s distance among *A. fumigatus* isolates from food sources. Each node represents a unique MLG, scaled by its frequency (number of isolates), and colored according to food origin. Edges represent genetic distances between MLGs, with thickness proportional to relatedness. The network illustrates overall genetic connectivity across substrates, with local clustering of certain food-associated genotypes.

The minimum spanning network (MSN) constructed from Bruvo’s distance identified 33 MLGs connected in a highly reticulated network (Figure 5). Nodes were generally well connected, indicating limited genetic structuring among food sources. Some degree of clustering by substrate was observed, particularly for genotypes from ground black pepper, but overall, the network suggested substantial gene flow or shared ancestry among populations. The size of the nodes, proportional to MLG frequency, highlighted a few dominant genotypes across food sources, coexisting with multiple low-frequency or singleton genotypes.

## 4. Discussion

Although no phenotypically resistant isolates were detected in this study, our findings reveal variations in susceptibility and the high genetic diversity of *A. fumigatus* in common food products, warranting a detailed discussion.

Based on the susceptibility breakpoints reported by Jørgensen et al. [57], which indicate a MIC_50_ ≤ 2 µg/mL for susceptible *A. fumigatus* strains, all isolates evaluated in the present study were classified as susceptible to tebuconazole. Among the triazoles tested, tebuconazole showed the greatest variability in EC_50_ values, followed by itraconazole, while voriconazole exhibited the least variation, with all isolates remaining within the susceptible range. The broader EC_50_ range observed for tebuconazole and itraconazole, particularly in isolates from plant-based substrates, may reflect the early stages of adaptive response to environmental exposure to the fungicide, as proposed by Harish et al. [48] under the concept of “triazole priming”.

Triazole priming refers to the repeated or prolonged exposure of *A. fumigatus* to subinhibitory concentrations of azole compounds, which can induce physiological or epigenetic changes that gradually increase fungal tolerance, even in the absence of genotypic resistance. In the case of tereré, although tebuconazole is not directly applied during cultivation, indirect exposure through environmental azole residues in soil, water, or air may exert selective pressure [48]. This scenario is supported by studies demonstrating that even non-agricultural environments or untreated crops may harbor *A. fumigatus* strains with early shifts in sensitivity profiles, potentially driven by fungicide drift or cohabitation with exposed microorganisms. For instance, Burks et al. [39] reported that resistant *A. fumigatus* strains can emerge in environments such as compost waste, contaminated soil, and sewage sludge, reinforcing the notion that azole selection pressure extends beyond direct agricultural applications.

This latent pressure is particularly concerning, given that our findings of susceptibility contrast with a previous environmental survey in Brazil, which reported varying levels of azole resistance, from low prevalence to concerning hotspots [58]. Although our isolates did not exhibit phenotypic resistance, the widespread use of tebuconazole in the analyzed crops warrants caution. This agricultural fungicide shares high structural homology with medical azoles, particularly itraconazole and voriconazole [34,59]. Recent experimental studies have demonstrated that in vitro exposure to tebuconazole can directly select for *A. fumigatus* isolates with reduced sensitivity to medical triazoles, even in the absence of the classic TR34/L98H mutation [41]. Therefore, the presence of the fungus in tebuconazole-treated dry foods represents a latent One Health risk, as continuous chemical pressure may eventually select for cross-resistant genotypes [38].

In this context, Brazil’s tropical agroecosystem poses a unique challenge for managing fungicide resistance, given year-round crop production, favorable climatic conditions for pathogen proliferation, and intensive fungicide use. According to Ceresini et al. [60], these conditions create a continuous selective environment that accelerates the evolution and spread of resistant fungal populations. The study emphasizes the importance of integrated resistance management strategies that combine chemical, biological, and cultural control practices while promoting rational fungicide use to safeguard both agricultural productivity and public health.

The potential source of this environmental pressure lies in the region’s widespread agricultural practices. Furthermore, in Brazil, 16 triazole active ingredients are currently registered for agricultural use. Of the commercial fungicide products containing triazoles, 30.8% (77 products) are based on tebuconazole, which is approved for use on over 80 different crops. Recommended application rates vary depending on the crop, target pathogen, and product formulation [61]. Although tebuconazole is not approved for use on crops such as black pepper and tereré, it is widely used in coffee cultivation to control coffee leaf rust (*Hemileia vastatrix*), one of the most severe diseases affecting coffee plants worldwide [62].

In coffee cultivation, the peak incidence of coffee leaf rust epidemics typically occurs during the fruiting and harvesting stages [63]. The most common control strategy relies on chemical treatments, particularly systemic fungicides such as triazoles [61]. Standard application rates for tebuconazole (e.g., approximately 193.5 g a.i./ha) typically result in field concentrations that could exceed the susceptibility thresholds for *A. fumigatus* [62]. While this concentration is sufficient to inhibit susceptible fungi, it may simultaneously impose selective pressure on native fungal populations, potentially driving the emergence of resistant strains even in crops where tebuconazole use is unauthorized.

From a One Health perspective, the application of tebuconazole in crops (even at sub-inhibitory concentrations) may create a shared chemical environment that could, in theory, link agricultural practices to clinical outcomes [38,39]. Since *A. fumigatus* is a ubiquitous saprophyte, inadvertent exposure to these agricultural azoles might select for cross-resistance to medical triazoles such as voriconazole and itraconazole, given their structural similarity [39,40,41]. Furthermore, previous studies have demonstrated that sub-lethal exposure to tebuconazole can induce “triazole priming”, facilitating adaptation to medical azoles [48]. Therefore, although the isolates in this study remained susceptible, the high genetic diversity observed suggests a dynamic population where resistance traits could possibly spread if selective pressure were to increase, potentially enabling these food chains to act as transmission vehicles for resistant pathogens to humans [44].

To contextualize these findings within a clinical framework, it is important to compare our results with established susceptibility breakpoints for medical triazoles. First introduced in 1990, itraconazole was reported to have *A. fumigatus* resistance in 1997, based on isolates collected in the late 1980s. Until 2004, however, such cases remained rare, particularly in the Netherlands. From then on, resistance rates began to rise, with subsequent data from 2008 and 2009 confirming this trend and shedding light on the underlying mechanisms of resistance [64]. Recent studies have demonstrated a broad range of EC_50_ values for *A. fumigatus* when exposed to itraconazole, reflecting variability in susceptibility depending on environmental versus clinical origin. For instance, Rybak et al. [65] reported EC_50_ values ranging from 0.5 to 4.0 µg/mL among clinical isolates. In contrast, a study conducted in Greece identified *A. fumigatus* in agricultural soils, with itraconazole EC_50_ values around 0.25 µg/mL; notably, a resistant isolate exhibited a minimum inhibitory concentration (MIC) greater than 8 µg/mL [66]. According to the European Committee on Antimicrobial Susceptibility Testing (EUCAST, version 10.0, 2020), the clinical breakpoint for itraconazole resistance in *A. fumigatus* is defined as MIC > 2.0 µg/mL. Isolates with MICs ≤ 1.0 µg/mL are considered susceptible, while those at 2.0 µg/mL are classified as intermediate [67]. Similarly, EUCAST also provides clinical breakpoints for voriconazole against *A. fumigatus*, with susceptibility defined as MIC ≤ 1 µg/mL and resistance as MIC > 2 µg/mL. An area of technical uncertainty (ATU) is defined at 2 µg/mL, with the epidemiological cutoff value (ECOFF) coinciding with the susceptibility threshold (MIC ≤ 1 µg/mL), delineating wild-type populations. It is important to note that while the agar-based screening method used in this study provides reliable EC50 values for surveillance, direct comparison with CLSI/EUCAST broth microdilution breakpoints requires caution.

The clinical relevance of these breakpoints is underscored by voriconazole’s role as primary therapy and the growing concern about reservoirs of resistance. Voriconazole is the first-line treatment for both chronic pulmonary aspergillosis (CPA) and invasive pulmonary aspergillosis (IPA), with therapeutic regimens typically lasting from 6 to 12 weeks for IPA and up to 6 months for CPA [28]. While clinical resistance to azoles in *A. fumigatus* isolates from humans is a growing concern, increasing evidence suggests that both domestic and wild animals may also serve as reservoirs for resistant strains. A systematic review of studies published between 2013 and 2024 identified non-wild-type (NWT) *A. fumigatus* isolates in dogs, cats, horses, birds, and zoo animals, with cases reported across multiple regions, including outside Europe. Itraconazole remains the most commonly used antifungal in companion animals and horses, whereas voriconazole is frequently administered to wildlife and zoo species. Reported MIC_50_ values for voriconazole in animal-derived *A. fumigatus* isolates range from 0.064 to 0.75 µg/mL, depending on the host species and clinical context [68]. These findings underscore the potential role of animals in the persistence and dissemination of azole-resistant *Aspergillus* strains.

Similar mutations have been reported in azole-resistant *A. fumigatus* (ARAf) strains lacking the common TR34/L98H or TR46/Y121F/T289A mutations. Although the M172V substitution alone is not associated with a resistant phenotype, it belongs to a group of mutations that, when present in combination with others, may contribute to reduced azole susceptibility and the development of resistance profiles [69].

This interplay between agricultural practices, environmental isolates, and mutations with potential clinical relevance highlights the importance of a broader perspective. Although azole resistance was not detected in the *A. fumigatus* isolates analyzed in this study, the implementation of a One Health approach remains essential for integrated surveillance and risk assessment. *A. fumigatus* is a widely distributed fungus that can develop resistance under selective pressures arising from agricultural, clinical, and veterinary practices. Continuous monitoring of this pathogen in food products is crucial, as contaminated commodities may serve as potential reservoirs for resistant strains, posing a threat to both human and animal health. This perspective aligns with United Nations Sustainable Development Goals 2 (Zero Hunger) and 3 (Good Health and Well-being) by emphasizing the critical intersection of food safety and public health. Implementing integrated pest management to minimize the selection of cross-resistant fungal strains is essential for sustainable food systems [38]. By adopting integrated pest management and reducing reliance on prophylactic azole use, agricultural sectors can protect crop yields without compromising the efficacy of critical medical antifungals [47]. This alignment with the One Health framework supports food security by maintaining effective disease control tools for future generations while simultaneously reducing the environmental reservoir of resistant genotypes that threaten human health, as emphasized in recent multi-agency regulatory reports [70].

Finally, the population-genetics data reveal another dimension of this issue, underscoring the diversity of fungal strains circulating in these products. Our analyses demonstrate that *A. fumigatus* populations associated with diverse food substrates exhibit substantial genotypic diversity (Figure 4 and Figure 5). The identification of 33 MLGs from 42 isolates highlights the coexistence of clonal expansion and unique genotypes, probably reflecting both asexual reproduction and ongoing diversification. The relative contribution of MLGs across substrates indicates that some food sources, such as ground black pepper, may harbor a more heterogeneous genotypic composition, potentially due to differences in ecological conditions, processing, or contamination sources.

The MSN (Figure 5) revealed limited population structuring among food sources, suggesting gene flow or common contamination origins across substrates. However, the partial clustering of genotypes within certain foods indicates localized amplification of specific clones. These findings are consistent with the epidemiological relevance of *A. fumigatus*, a ubiquitous saprotroph with high dispersal capacity. Importantly, the diversity observed in food-associated isolates parallels patterns reported in clinical and environmental populations, underscoring the potential role of foods as reservoirs for genetically diverse strains that may also circulate in human environments.

## Figures and Tables

**Table 1:** Samples and Origins of Commercial Products Used for *A. fumigatus* Isolation.

Sample ID	Product	Physical Form	Origin
1	Black pepper	Grain	Estrela D’Oeste, SP *
2	Black pepper	Grain	Ilha Solteira, SP *
3	Black pepper	Grain	Mirassol, SP *
4	Black pepper	Grain	Neves Paulista, SP *
5	Black pepper	Ground	Mirassol, SP *
6	Black pepper	Ground	Neves Paulista, SP *
7	Black pepper	Ground	Pouso Alegre, MG *
8	Black pepper	Ground	Estrela D’Oeste, SP *
9	Black pepper	Ground	Ilha Solteira, SP *
10	Black pepper	Ground	Campo Grande, MS *
11	Black pepper	Ground	Neves Paulista, SP *
12	Green coffee	Grain	Araxá, MG
13	Green coffee	Grain	Araxá, MG
14	Green coffee	Grain	Araxá, MG
15	Green coffee	Grain	Paraguaçu, MG
16	Green coffee	Grain	Paraguaçu, MG
17	Yerba mate	Chopped	Almirante Tamandaré, PR
18	Yerba mate	Chopped	Canoinhas, SC
19	Yerba mate	Chopped	Santo Antônio do Sudoeste, PR
20	Yerba mate	Chopped	Naviraí, MS
21	Yerba mate	Chopped	Chapecó, SC
22	Yerba mate	Chopped	Canoinha, SC
23	Yerba mate	Chopped	Laranjeiras do Sul, PR
24	Yerba mate	Chopped	Campo Grande, MS
25	Yerba mate	Chopped	Ponta Porã, MS

* Black pepper is usually produced in Bahia, Espirito Santo, and Pará regions; however, the samples purchased only had the identification of the distribution center [49].

**Table 2:** Primers used for molecular identification of *A. fumigatus* and *Aspergillus* section Fumigati [52].

Genus/Species	Target	*Primers* (5′-3′)		Length
*Aspergillus* section Fumigati	*β-tubulin*	F	AGGCAGACCATCTCTGGTGAG	153 bp
		R	TCGGAGGAGCCATTGTAGC	
	*rodlet A*	F	CCAGGCTCAGCTCTCTTGCT	105 bp
		R	CCACCACCGATGAGGTTCTT	
*Aspergillus fumigatus*	*β-tubulin*	F	TGACGGGTGATTGGGATCTC	198 bp
		R	CGTCCGCTTCTTCCTTGTTT	
	*rodlet A*	F	ACATTGACGAGGGCATCCTT	313 bp
		R	ATGAGGGAACCGCTCTGATG	

## Data Availability

The original contributions presented in this study are included in the article/Supplementary Materials. Further inquiries can be directed to the corresponding author.

## Supplementary Materials

The following supporting information can be downloaded at: https://www.mdpi.com/article/10.3390/jof12010072/s1, Figure S1. Fungal growth and colonization of *Aspergillus fumigatus* on food substrates (green coffee) after incubation. Figure S2. Whole-grain food samples evaluated in the study: green coffee, roasted coffee, black pepper, and yerba mate. Figure S3. Sensitivity of *Aspergillus fumigatus* isolates to tebuconazole at different concentrations. Figure S4. Sensitivity of *Aspergillus fumigatus* isolates to itraconazole at different concentrations. Figure S5. Sensitivity of *Aspergillus fumigatus* isolates to voriconazole at different concentrations. Table S1. Primers used for PCR amplification and sequencing of the *Aspergillus fumigatus CYP51A* gene. Table S2. Summary of the amplification primers used for selected STR loci, including repeat features and discriminatory indices. Table S3. Summary of *Aspergillus fumigatus* isolates and multilocus genotypes (MLGs) across food substrates. Number of isolates, unique multilocus genotypes (MLGs), clonal diversity (MLG/isolates) detected per substrate and product sizes (bp) are presented for STRAf markers 2A, 2B, 2C, 3A, 3B, 4A, 4B and 4C.
