# Supplementary material for: A One Health Perspective on Aspergillus fumigatus in Brazilian Dry Foods: High Genetic Diversity and Azole Susceptibility
Source: J Fungi (Basel). 2026 Jan 16;12(1):72. doi: 10.3390/jof12010072 (PMC12843012; doi:10.3390/jof12010072)
Supplement: Supplementary file 1 [file jof-12-00072-s001.zip › Supl Tables.pdf]

Table S1. Primers used for PCR amplification and sequencing of the *Aspergillus fumigatus* *CYP51A* gene.

Target	Primers	Annealing temperature (°C)	Amplicon size (bp)
CYP51A	51AF1: GGAGAAGGAAAGAAGCACTCT<br>51AR1: CTGTCTCACTTGGATGTG<br>51AFS: CCTCTATCATGACCTGGACA<br>51ARS: ATGAGCAGCATCTCGCTTCT	53	2040

Table S2. Summary of the amplification primers used for selected STR loci, including repeat features and discriminatory indices.

Primer name	Forward primer sequence[1, 2]	Reverse primer sequence[1]	Repeat unit
STR*Af* 2A	AAGGGTTATGGCCATTAGGG	GACCTCCAGGCAAAATGAGA	GA
STR*Af* 2B	TATTGGATCTGCTCCCAAGC	GAGATCATGCCCAAGGATGT	AG
STR*Af* 2C	TCGGAGTAGTTGCAGGAAGG	AACGCGTCCTAGAATGTTGC	CA
STR*Af* 3A	GCTTCGTAGAGCGGAATCAC	GTACCGCTGCAAAGGACAGT	TCT
STR*Af* 3B	CAACTTGGTGTCAGCGAAGA	GAGGTACCACAACACAGCACA	AAG
STR*Af* 4A	TTGTTGGCCGCTTTTACTTC	GACCCAGCGCCTATAAATCA	TTCT
STR*Af* 4B	CGTAGTGACCTGAGCCTTCA	GGAAGGCTGTACCGTCAATCT	CTAT
STR*Af* 4C	CATATTGGGAAACCCACTCG	ACCAACCCATCCAATTCGTAA	ATGT
FAM_M13	TGTAAAACGACGGCCAGT	-	-
TET_M13	TGTAAAACGACGGCCAGT	-	-
CY3_M13	TGTAAAACGACGGCCAGT	-	-
CY5_M13	TGTAAAACGACGGCCAGT	-	-

[1] All primer sequences are given in the 5’ to 3’ direction.
[2] Except for the fluorophores, all forward primer sequences were synthesized with the universal M13 sequence (TGTAAAACGACGGCCAGT) at their 5′ end.

Table S3. Summary of *Aspergillus fumigatus* isolates and multilocus genotypes (MLGs) across food substrates. Number of isolates, unique multilocus genotypes (MLGs), genetic diversity (MLG/isolates) detected per substrate and product sizes (bp) are presented for STRAf markers 2A, 2B, 2C, 3A, 3B, 4A, 4B and 4C.

MLG	Isolate	Food source	2A	2B	2C	3A	3B	4A	4B	4C
1	A11P1-2_2	Ground black pepper	182	182		205	199	182	180	199
2	A11P1V_2	Ground black pepper	172	172		187	181	182	180	208
3	A11P4-2_2	Ground black pepper	172	172		202	208	254	248	208
4	A6P3-3_2	Ground black pepper	160	198	159	205	202	202	196	208
5	A4G3-4_2	Whole black pepper	160	176	161	160	202	202	196	232
5	A4G3-2_2	Whole black pepper	160	176	161	160	202	202	196	232
6	A11P51_2	Ground black pepper	160	174	161	160	202	202	196	
7	A4G4-3_2	Whole black pepper	188	188	153	178	181	182	180	196
8	A4G7-2cd_2	Whole black pepper	162	170	163	163	181	182	180	211
9	A9P1-1_2	Ground black pepper	162		163	163	202	202	196	223
10	A3G4cd_2	Whole black pepper	162		153	196	220	202	200	199
11	A4G6-2_2	Whole black pepper	190	190	153	193	181	182	180	208
12	A6MF2_2	Yerba mate	190	192	151	193	193	194	192	208
13	A4G4cd_2	Whole black pepper	176	176	161	160	202	202	196	232
14	A3MVF1_2	Yerba mate	176	176	159	205	193	194	180	208
15	A4G13V_2	Whole black pepper	176	176	153	202	202	202	196	202
16	A9MV8F1_2	Yerba mate	176	176	153	178	193	194	192	232
16	A9MF2_2	Yerba mate	176	176	153	178	193	194	192	232
17	A4G5_2	Whole black pepper	184	184	153	199	202	202	192	223
18	A3G6V1_2	Whole black pepper	184	184	153	199	202	202	196	223
19	A1C2-1_2	Green coffee	184	186	153	184	181	182	164	217
20	A4G6-1_2	Whole black pepper	184	186	153	193	181	182	164	217
21	A3G9_2	Whole black pepper	174	174	153	193	202	202	180	223
22	A3G9-2_2	Whole black pepper	174	174	153	193	202	202	192	223
23	A3C3_2	Green coffee	174	174	153	193	202	202	196	223
24	A4G4-1V_2	Whole black pepper	174	174	153		202	202	200	223
25	A6P4V_2	Ground black pepper	174	174		196	220	218	212	208
26	A9P5V_2	Ground black pepper	186	186	153	199	181	182	180	199
27	A4G4-2_2	Whole black pepper	186	186	153	205	202	202	180	208
28	A4G3-1_2	Whole black pepper	186	186	153	205	202	202	196	208
29	A3C6_2	Green coffee	186	186		193	181	182	164	217
30	A9P3-1_2	Ground black pepper	180	180	165		202	202	196	223
31	A3G3V_2	Whole black pepper	180	180	159	196	193	194	188	220
32	A3G32V_2	Whole black pepper	180	180	159	196	193	194	192	220
32	A3G3V1_2	Whole black pepper	180	180	159	196	193	194	192	220
33	A5C5-3_2	Green coffee	180	180	153	193	202	202	196	208
33	A5C5-2_2	Green coffee	180	180	153	193	202	202	196	208
33	A5C5-1_2	Green coffee	180	180	153	193	202	202	196	208
33	A1C2-2_2	Green coffee	180	180	153	193	202	202	196	208
33	A1C1_2	Green coffee	180	180	153	193	202	202	196	208
33	A1C1-2_2	Green coffee	180	180	153	193	202	202	196	208
33	A9MF1_2	Yerba mate	180	180	153	193	202	202	196	208
