# Supplementary figures and images for: A One Health Perspective on Aspergillus fumigatus in Brazilian Dry Foods: High Genetic Diversity and Azole Susceptibility
Source: J Fungi (Basel). 2026 Jan 16;12(1):72. doi: 10.3390/jof12010072 (PMC12843012; doi:10.3390/jof12010072)

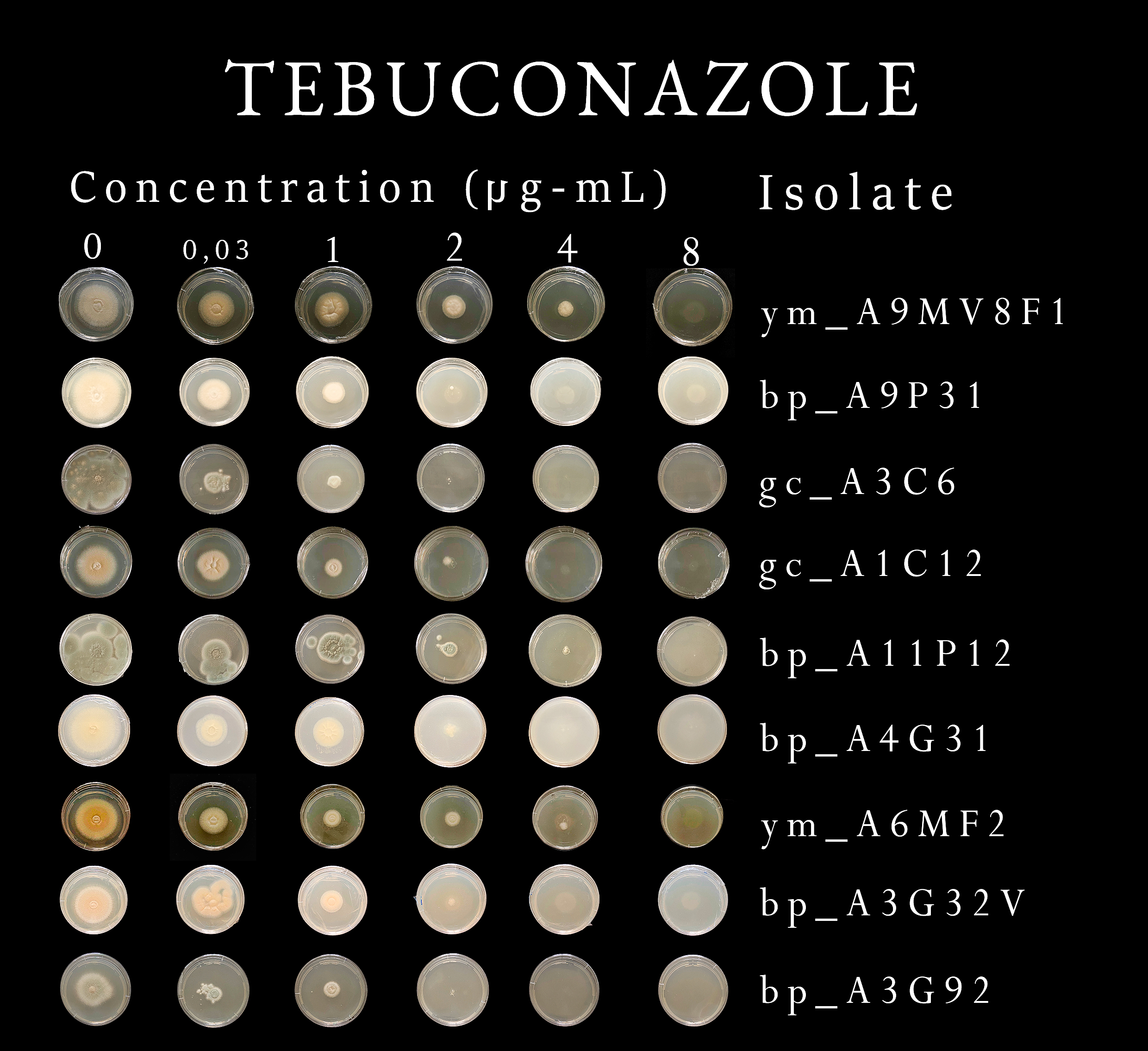

Supplement: Supplementary file 1 [file jof-12-00072-s001.zip › Figure S3 (Tebuconazole).JPG]

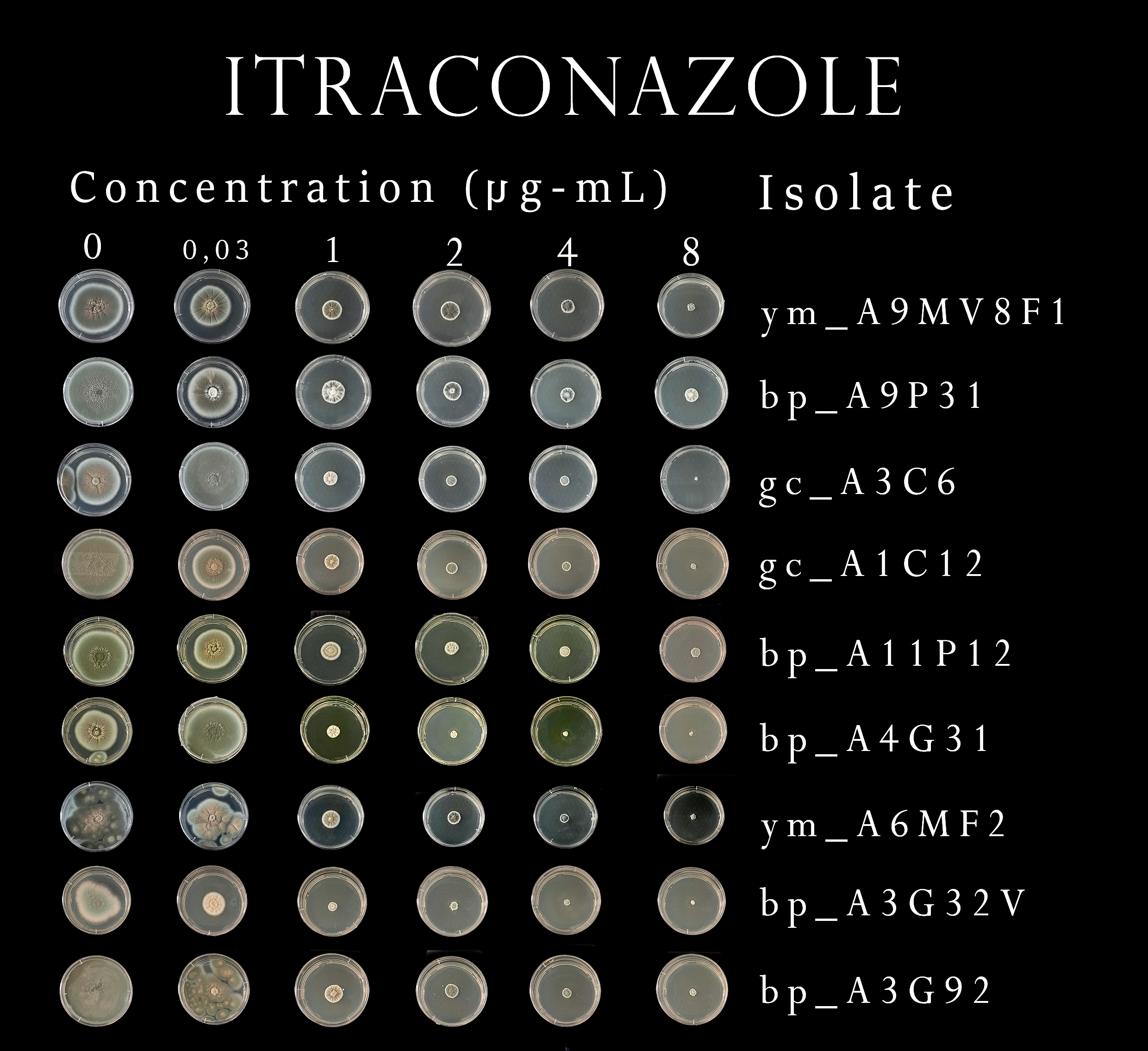

Supplement: Supplementary file 1 [file jof-12-00072-s001.zip › Figure S4 (Itraconazole).jpg]

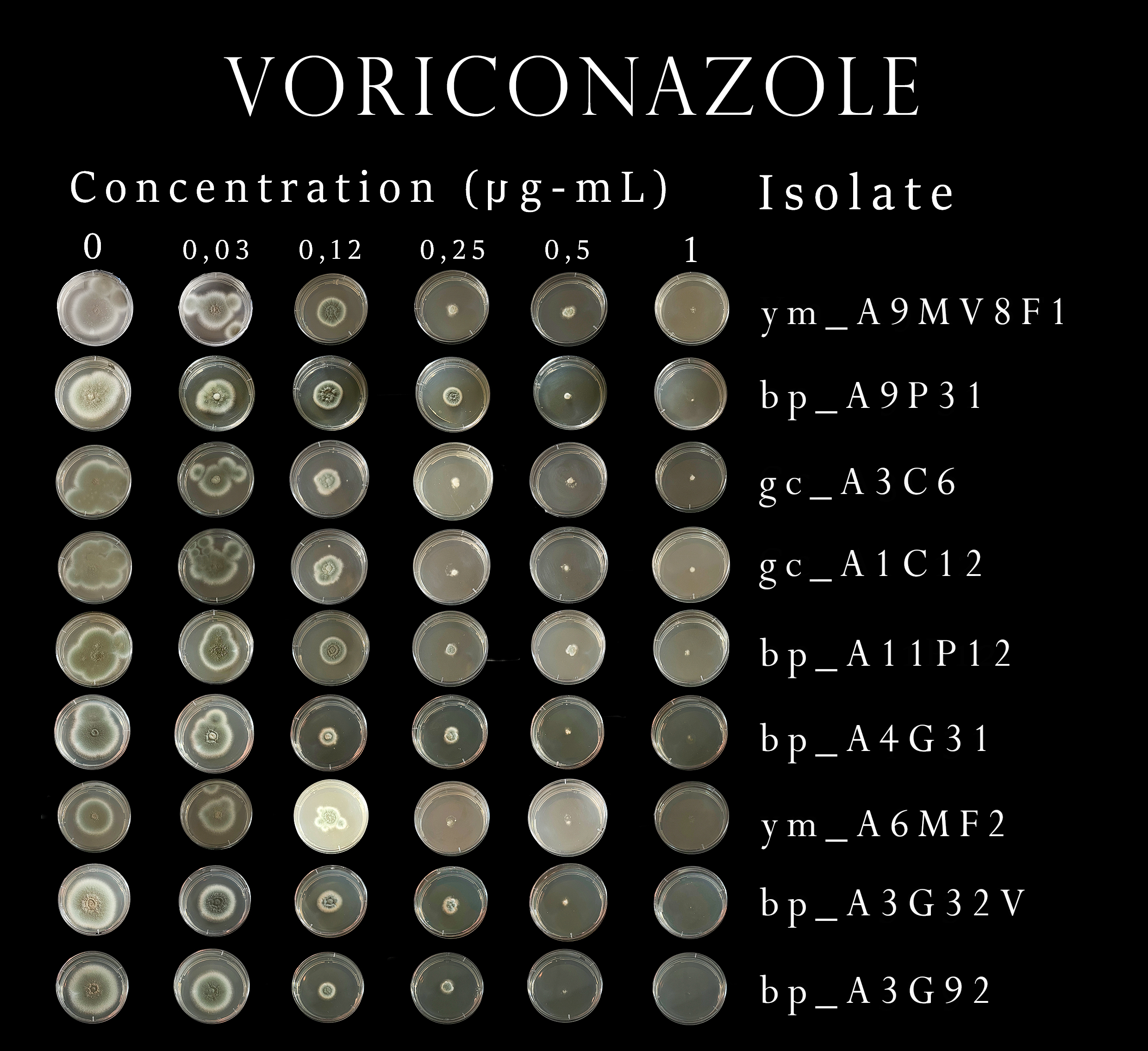

Supplement: Supplementary file 1 [file jof-12-00072-s001.zip › Figure S5 (Voriconazole).jpg]

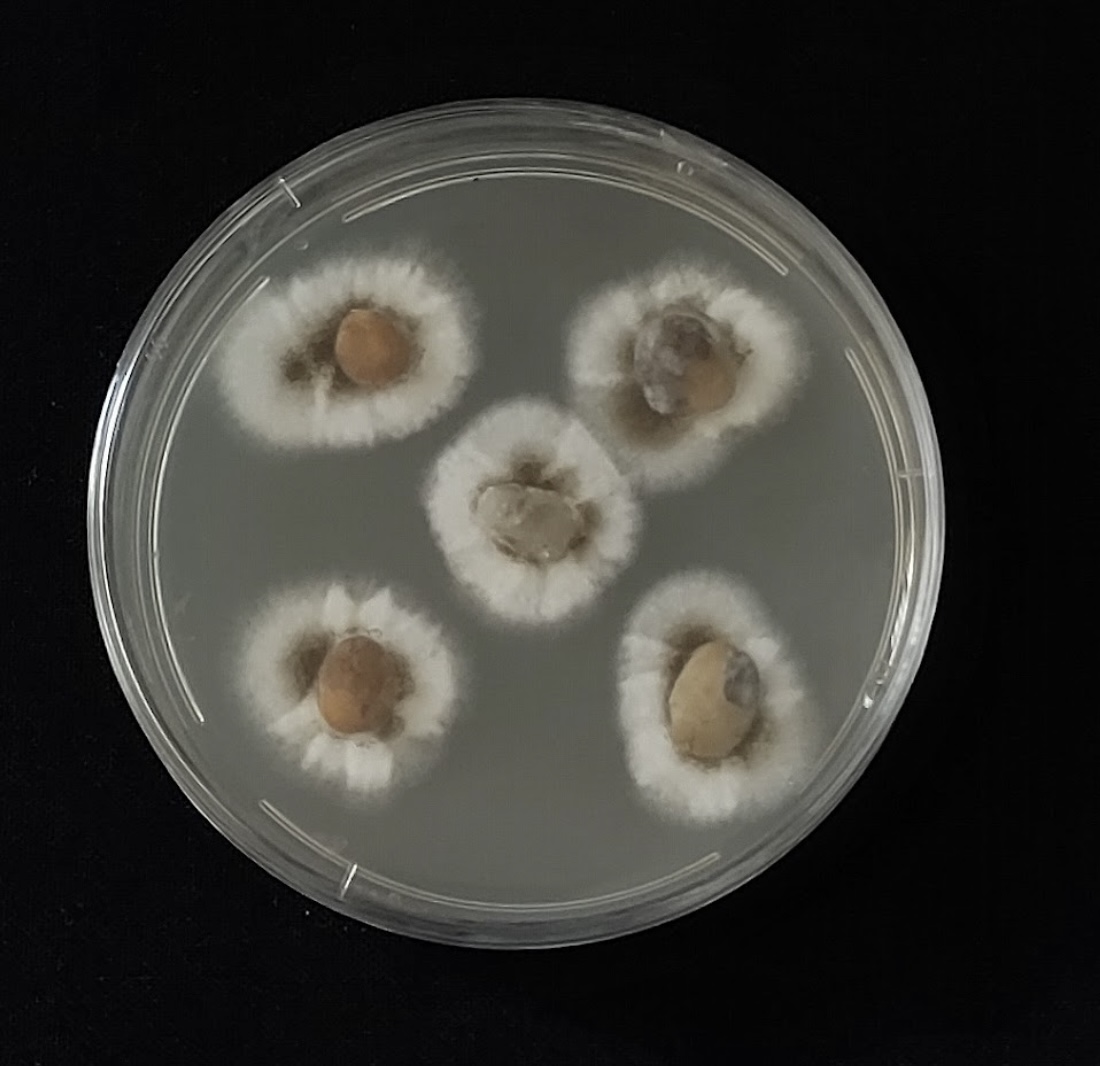

Supplement: Supplementary file 1 [file jof-12-00072-s001.zip › Figure S1. Food Samples.jpg]

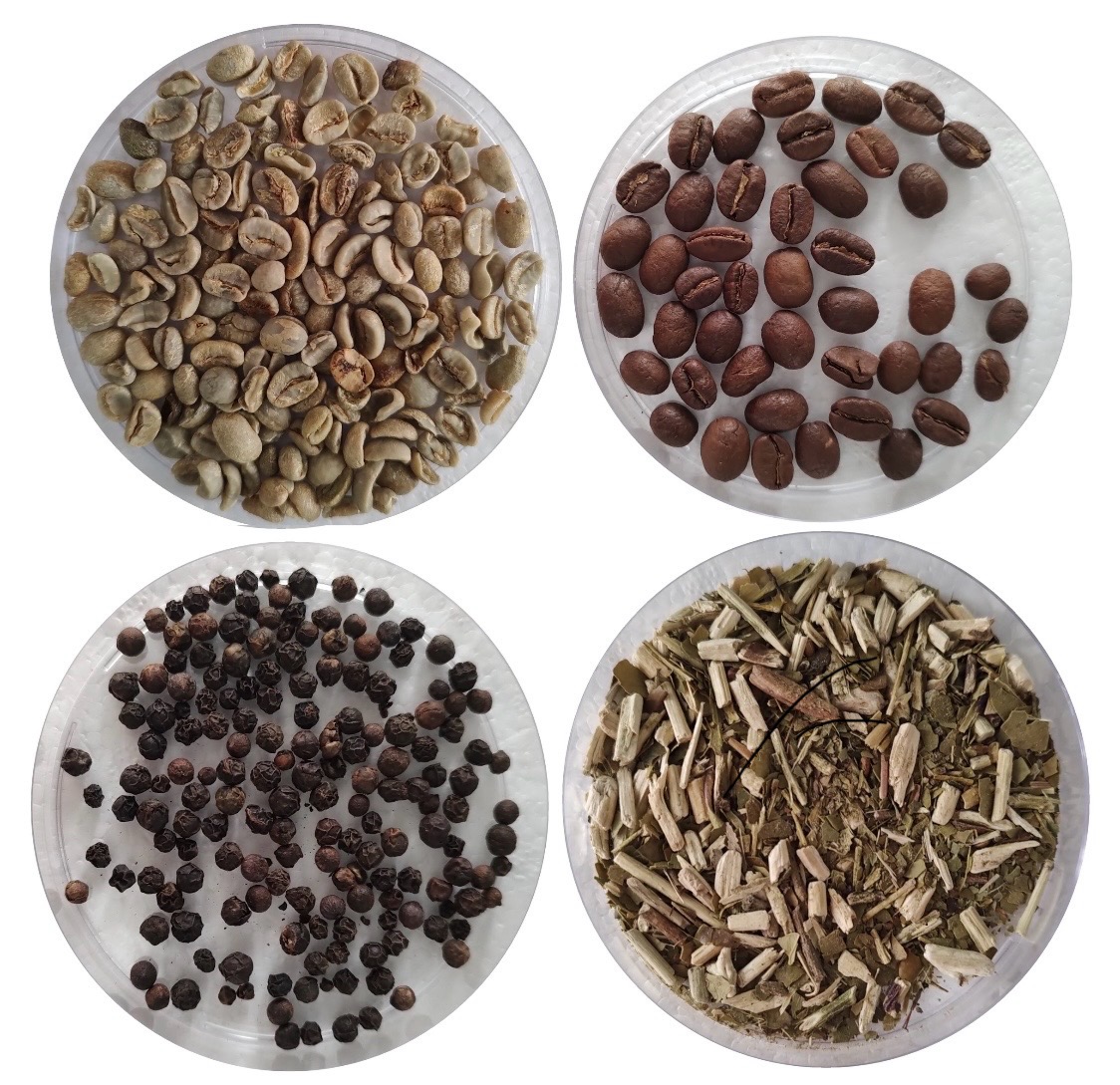

Supplement: Supplementary file 1 [file jof-12-00072-s001.zip › Figure S2. whole-grain samples.jpg]
